# Post-operative delirium in the patient with hip fracture: The journey from hospital arrival to discharge

**DOI:** 10.3389/fmed.2022.1080253

**Published:** 2022-11-23

**Authors:** Danielle Ní Chróinín, Alwin Chuan

**Affiliations:** ^1^Liverpool Hospital, Liverpool, NSW, Australia; ^2^South Western Sydney Clinical School, UNSW Sydney, Liverpool, NSW, Australia; ^3^Ingham Institute of Applied Medical Research, Liverpool, NSW, Australia

**Keywords:** delirium, post-operative, hip fracture, prevention, multidisciplinary, acute confusional state, cognitive impairment

## Abstract

Delirium- an acute disorder of attention and cognition- is the commonest complication following hip fracture. Patients with hip fracture are particularly vulnerable to delirium, and many of the lessons from the care of the patient with hip fracture will extend to other surgical cohorts. Prevention and management of delirium for patients presenting with hip fracture, extending along a continuum from arrival through to the post-operative setting. Best practice guidelines emphasize multidisciplinary care including management by an orthogeriatric service, regular delirium screening, and multimodal interventions. The evidence base for prevention is strongest in terms of multifaceted interventions, while once delirium has set in, early recognition and identification of the cause are key. Integration of effective strategies is often suboptimal, and may be supported by approaches such as interactive teaching methodologies, routine feedback, and clear protocol dissemination. Partnering with patients and carers will support person centered care, improve patient experiences, and may improve outcomes. Ongoing work needs to focus on implementing recognized best practice, in order to minimize the health, social and economic costs of delirium.

## Main body

“He’s been up all night. He was terrified.” The patient’s daughter looked as tired as her father, who was snoring now in his bed on our orthopedic ward. Further questioning revealed an exhausting night of agitation, attempted climbing from bed, hallucinations, and intravenous catheters being pulled out by the distressed patient. Despite the brightness of the sunlight streaming through the window, he was now barely rousable, grunting when moved, and had missed his breakfast and morning oral medications.

Sadly, this picture of delirium- an acute disorder of attention and cognition- is common on orthopedic and other hospital wards. The journey of a patient with hip fracture in many ways epitomizes that of the surgical patient at risk of delirium. These patients are older, have fallen, are often frail, and many have underlying cognitive impairment. Here in Australia, almost 1 in 3 is resident in a residential aged care facility (nursing home) even prior to surgery (1), and the recognized 30–40% with existing dementia or cognitive impairment likely under-estimates the true number, due to suboptimal assessment (2). Patients with underlying cognitive impairment are 3 times more likely to sustain a hip fracture than those without (3), and are at increased risk of delirium (4, 5). Rates of delirium vary, with reports of up to 65% of patients experiencing delirium following hip fracture (6). It is the commonest complication following hip fracture, yet more than 30% of delirium is likely to be preventable, including in hip fracture cohorts (4, 7, 8), making it a critical and attainable target. Nonetheless, the pathophysiology of delirium, in the post-operative context or otherwise- remains poorly understood, with neuro-inflammation and cerebral metabolic insufficiency being the two most-favored theories explaining the predisposing and trigger events leading to delirium onset (8, 9).

The negative sequelae of delirium- and thus the potential advantages to delirium prevention- encompass the patient, hospital, and society. Delirium is commonly associated with increased hospital LOS, in multiple studies of hip fracture and other surgical/trauma patients, including in a recently published large retrospective analysis of > 4,000 patients from the Australian and New Zealand Hip Fracture Registry (ANZHFR) (10). A US paper noted that delirium was one of the two most notable predictors of prolonged LOS in hip fracture patients (the other being delayed time to surgery) (11). Delirium is also associated with increased risk of in-patient falls, mortality, and future risk of dementia, and with significant distress for patients and family members (12–15). The total costs of delirium in Australia, however, extend beyond easily-captured health system costs; including other financial costs (such as productivity costs, informal care) and that associated with burden of disease and loss of well-being, total annual cost has been estimated to be in the order of AUD$8.8 billion (2016–2017 data) (16), which, with 132,595 occurrences of delirium in Australia alone each year, would mean that each occurrence of delirium would cost approximately AUD$66,000.

Delirium prevention begins at the time of the patient’s arrival. Early orthogeriatrician input and comprehensive geriatric assessment are likely to improve outcomes, including reducing delirium incidence and severity (17–20). Best practice guidelines such as the Australian Hip Fracture Care Clinical Care Standard highlight that care at presentation, in addition to diagnostics, should include pain control, assessment of medical reasons for the fall, exclusion of other injuries- including head injury- and specifically highlight the need to “screen for cognitive impairment and risk factors for delirium and put in place interventions to prevent delirium based on this assessment” (21). Comprehensive geriatric assessment commonly incorporates assessment of medical (co-)morbidity, drugs and polypharmacy, cognition, mobility, function, social circumstances, and establishing goals of care. Such assessment is potentially associated with reduced post-operative delirium, improved delirium diagnosis, and reduction in other post-operative complications (17, 22, 23), which may contribute to delirium. Ideally, such an orthogeriatric approach to care should be instituted from admission.

Existing evidence-based guidelines and recommendations note the importance of early cognitive assessment, delirium risk screening and prevention of ‘preventable’ delirium (2, 21, 24). Yet data consistently highlight that cognitive screening amongst patients with hip fracture remains sub-optimal (2, 25). Tools such as the Delirium Risk Assessment Tool can help identify patients at highest risk (26, 27), and facilitate focused risk management plans. In addition to this, validated screening instruments [e.g., 4AT, Confusion Assessment Methods (CAM)] should be employed to assist in detection, in conjunction with appropriated cognitive screening tools where needed (28–30). Specific patient cohorts, such as those from culturally or linguistically diverse backgrounds, or Aboriginal and Indigenous patients, may benefit from culturally appropriate cognitive assessment (31–33). In patients who are unwell or cognitively impaired, the need for a collateral history is paramount (34, 35). However, evidence suggests that collateral history from family/carer is often neglected- being either absent or sparse- with Fitzpatrick et al. (34) highlighting that it is “alarming that such an essential component of clinical assessment is so often disregarded.” In addition to screening for and recognition of delirium, proactive avoidance of triggers- such as constipation, urinary retention and catheterization and deliriogenic medications- and timely pain assessment and management are key in the patient with hip fracture (21, 36, 37). The benefit of addressing delirium risk factors is highlighted by multifaceted interventions which target these risks. For example, data from the wider in-patient population have shown reduced delirium incidence- and other adverse outcomes such as falls- with the Hospital Elder Life Program, which focuses on cognitive impairment, sleep deprivation, immobilization, visual impairment, hearing impairment and dehydration (38, 39).

Despite guidelines recommending early pain assessment (18, 19), early and repeated pain assessment remains suboptimal (2, 40). Along with regular assessment, pain control may require a multimodal approach from amongst simple analgesics, anti-inflammatories in appropriate patients, opiates with appropriate monitoring, and regional nerve blocks such as fascia-iliaca block (FIB) (19, 41). Analgesic-centric multicomponent bundles of care have been associated with reductions in early post-operative delirium amongst patients with hip fracture (42).

In contrast to the relatively strong evidence for addressing delirium risk factors either side of surgery, intra-operative factors have been less convincingly associated with post-operative delirium. In the setting of an imperfect evidence base, regional anesthesia does not seem to confer any delirium-reduction benefits over general anesthetic, although potential advantages in terms of adverse events, hospital length-of-view and even mobilization were identified in a small number of studies (43, 44). Duration of surgery may be associated with increased risk (45). Similarly, despite theoretical advantages, lighter sedation during surgery has not been convincingly associated with delirium risk (46), and the question of whether higher-dose propofol might contribute to post-operative delirium risk remains unanswered (47). On the other hand, the need for intensive care and/or ventilator care post-operatively are likely to increase risk, as demonstrated by a large Brazilian study of almost 60,000 hip fracture patients who underwent regional anesthesia (48).

The principles of pre-operative delirium prevention, assessment and management extend to the post-operative phase. Early, coordinated orthogeriatric and multidisciplinary input aims to reduce post-operative complications in addition to providing benefits regarding care coordination. Nurses will play a vital role in the post-operative phase of the patient’s recovery. Factors which will continue to impact on delirium risk in this post-operative will include management of pain, minimizing polypharmacy, rationalizing medications, and avoiding (or planned earliest removal of) invasive devices such as urinary catheters (4, 38, 49, 50). Given the fluctuant course of delirium, and the prolonged post-operative risks for same in hip fracture cohorts, patients should be monitored regularly for cognitive, behavioral, and clinical deterioration; some guidelines suggest that all in-patients should be assessed at least daily (51). The regular assessment of pain assessment and effective analgesic management are critical to post-operative care (21), both to improve the patient’s quality of life and comfort, and reduce delirium risk. For patients with cognitive impairment/dementia, many tools have been developed. Tools such as the Faces Pain Scale, Abbey Pain Scale and Pain Assessment in Advanced Dementia (PAINAD), Pain Assessment Checklist for Seniors with Limited Ability to Communicate and Mobilization-Observation-Behavior-Intensity-Dementia (MOBID) scale may be useful in such cohorts (52). Pain management is likely to require a multifaceted approach, including systemic analgesia using paracetamol and short-acting opioid analgesics, aiming to minimize dosage and duration so as to reduce opioid-associated harm (53). A systematic review (2011) has previously indicated that the evidence for strategies such as acupressure, relaxation therapy, transcutaneous electrical neurostimulation, and physical therapy regimens, is inconclusive (54).

Early mobilization is also promoted for patients following hip fracture, to promote recovery of mobility and function and mobility. Mobility is also promoted for patients in terms of delirium prevention (51). However, its potential relationship to delirium prevention in hip fracture patients is complex. Nonetheless, a recent study highlighted that those who mobilized early post-operatively had reduced (post-operative) delirium compared to those who remained bedbound (55), and delirium itself can often be a barrier to early mobilization (55, 56). Early mobilization is associated with improved likelihood of discharge by 30 days, irrespective of delirium status (57).

In patients who do develop delirium, management must focus on the identification of the cause, as well as prevention of complications, such as functional decline, dehydration, malnutrition, falls and pressure injuries, based on their risk (26). Partnering with patients and carers will support person centered care, improve patient experiences, and may improve outcomes (58, 59).

Principles which support a multidisciplinary approach to delirium prevention and pain management need to be embedded in local pathways for the care of patients with hip fracture, complemented by audit and quality improvement initiatives (18, 19). Strategies such as bundles of care have been associated with improved compliance with recommended delirium-reducing strategies (42), clinician support for their implementation (60), as well as with direct benefits in terms of incidence of post-operative delirium (9, 42). Yet the integration of models of care to support best practice is often suboptimal (61). Potential enablers of such integration might, for example, include interactive teaching methodologies, routine individualized feedback, and clear protocol dissemination (60, 62–66), while barriers may include educational deficits, lack of motivation at individual or institutional levels, environmental factors, and specific health professional characteristics such as age, sex or experience (67, 68). Translation of evidence into practice will need to account for local factors specific to the individual setting and local population. Furthermore, newer educational methods such as the “flipped classroom” and “train the trainer” approaches may enhance delirium learning for healthcare professionals, with a study by Sockalingam et al. (65) showing persistent benefit in delirium knowledge and delirium care self-efficacy at 6 months following institution of these strategies, and a mixed-methods study identifying that a delirium simulation-based flipped classroom approach “promoted higher level learning and engagement in inter-professional collaborative practice” (66). Hunter et al note that “dynamic, responsive implementation strategies and accessible educational modalities, which are flexible to needs of individual multidisciplinary team members and adapted to specific settings, will likely prove the most successful approach to adoption of evidence-based protocols [supporting hip fracture care]” (60).

## Conclusion

In conclusion, patients presenting to hospital with hip fractures are at high risk of developing delirium. Patients who do develop delirium suffer significantly worse outcomes including death and morbidity, and delirium leads to increased suffering to carers and family, and imposes a large financial burden on the health system. Best practice guidelines emphasize multidisciplinary care including management by an orthogeriatric service, regular delirium screening, and multimodal interventions. Up to 30% of hip fracture-related delirium may be preventable with this approach. The health, social and economic burden of delirium underscore the need for research focusing on reducing the incidence and impact of delirium in our patients with hip fracture.

## Author contributions

DN and AC were responsible for drafting, editing, and finalization of the manuscript. Both authors agreed to be accountable for the content of the work, contributed to the article and approved the submitted version.
